# Cross-Sectoral Big Data: The Application of an Ethics Framework for Big Data in Health and Research

**DOI:** 10.1007/s41649-019-00093-3

**Published:** 2019-10-01

**Authors:** Graeme T. Laurie

**Affiliations:** School of Law and JK Mason Institute for Medicine, Life Sciences and the Law, University of Edinburgh, Edinburgh, UK

**Keywords:** Big data, Cross-sectoral, Smart cities, Data linkage, Big data ethics

## Abstract

Discussion of uses of biomedical data often proceeds on the assumption that the data are generated and shared solely or largely within the health sector. However, this assumption must be challenged because increasingly large amounts of health and well-being data are being gathered and deployed in cross-sectoral contexts such as social media and through the internet of (medical) things and wearable devices. Cross-sectoral sharing of data thus refers to the generation, use and linkage of biomedical data beyond the health sector. This paper considers the challenges that arise from this phenomenon. If we are to benefit fully, it is important to consider which ethical values are at stake and to reflect on ways to resolve emerging ethical issues across ecosystems where values, laws and cultures might be quite distinct. In considering such issues, this paper applies the deliberative balancing approach of the *Ethics Framework for Big Data in Health and Research* (Xafis et al. 2019) to the domain of cross-sectoral big data. Please refer to that article for more information on how this framework is to be used, including a full explanation of the key values involved and the balancing approach used in the case study at the end.

## Background

This paper will illustrate the ethical and governance challenges that arise when biomedical data are generated, linked and/or shared in data environments beyond the health sector.

It is clear that any distinction between health and other types of data is becoming increasingly blurred as notions of health and well-being expand into multiples areas of citizens’ lives, and it is reasonable to assert that the promises of big data will only be fully realized if data can be safely and effectively linked and shared across a range of sources to address multiple objectives in the name of maximizing benefit. While such benefit can include addressing unmet health and social care needs, it can also encompass improvements in efficiency of services and reduction in costs. This is what is meant by ‘cross-sectoral’ sharing and use of data in this Framework, and it can include access to social media data. Examples include the following:

- *Google Flu Trends*, which was launched in 2008—after joint research with the US Centers for Disease Control (CDC)—to anticipate disease patterns based on algorithm monitoring of flu-related internet searches, combined with computer modelling. Early successes matched CDC surveillance systems and on a shorter timescale. However, the system has by no means been infallible, with under-reporting of H1N1 (swine flu) pandemic in 2009, and a discrepancy of almost double CDC estimates for flu in 2011–2012 (Butler 2013; Lazer et al. 2014). This is part of a wider phenomenon known as digital disease detection (DDD), and there are multiple examples, including the use of Twitter (Denecke 2017).
- *Corporate Wellness Programs* in the USA, which have been used by firms such as Walmart and Target to employ private companies to mine employees’ information to determine who might be at risk of diseases such as diabetes or which women might be pregnant (Ajunwa et al. 2016). The often-stated objective is then to nudge employees towards certain health-related services, with obvious downstream savings for employers. Financial incentives are frequently offered to participate, and it was estimated in 2016 that over 500 vendors were selling programs worth in excess of $6 billion annually.
- *The Administrative Data Research Network (ADRN)*, funded by the UK’s Economic and Social Research Council from 2013 to 2018, which was an initiative to organize an infrastructure allowing social researchers to use administrative data in safe and secure settings. Building on governance successes in the health sector such as the Farr Institute (see also Jutte et al. 2011), the ADRN worked in partnership with public bodies such as local authorities, the Department of Work & Pensions and the Department of Education ‘to provide a sound base for policy makers to decide how to tackle a range of complex social, economic, environmental and health issues’. Approved projects included (i) evaluating variation in special educational needs provision for children with Down syndrome (see also Downs et al. 2017) and (ii) analysing the relationships between health and homelessness services. The Network was established to provide a single point of access for researchers and to offer ‘interoperable governance’ mechanisms for data holding stakeholders to adopt to make available datasets for cross-sectoral sharing (Laurie and Stevens 2016).

Big datasets are potentially enriched further by the Internet of Things (IoT): the network of mobile phones, apps and wearable devices (such as ‘fitbits’), as well as home sensors, home appliances, and homes and cities themselves (the so called ‘smart’ homes and ‘smart cities’). These technologies enable streams of data about citizens’ behaviours to be shared and exchanged. Examples relating to improved health and welfare outcomes include:

- The *tracking of mobile phones* to monitor mass movement of over half of a million people out of Haiti’s capital, Port-au-Prince, after the 2010 earthquake. This real-time data was used to target relief programmes where they were most needed (Bengtsson et al. 2011).
- The McKinsey Global Institute 2018 report on *Smart Cities* (McKinsey Global Institute 2018), which highlights Singapore as one of the top five global cities with the most advanced technology base and among the top six in terms of the greatest number of applications adopted across a range of domains. Health is identified as one of eight areas where smart technologies will bring considerable benefits, and it is claimed that these factors have set Singapore on the path towards a ‘virtuous cycle of benefits’ that could result in an 8–15% lower disease burden.
- The *Internet of (Medical) Things* (IoMT), which refers to smart and wearable devices, apps and other technologies that capture or generate and/or share health and health-related data from citizens, normally with their own well-being as the primary objective. However, because of the volume and fluidity of data generated and the general lack of specific regulation across sectors, there is a current lack of deep understanding about what information is generated, used and shared and for what purposes.^1^ A 2017 network analysis of health and fitness apps showed, for example, that aggregate consumer data was often being shared within app ‘families’ and their partners (Grundy et al. 2017).

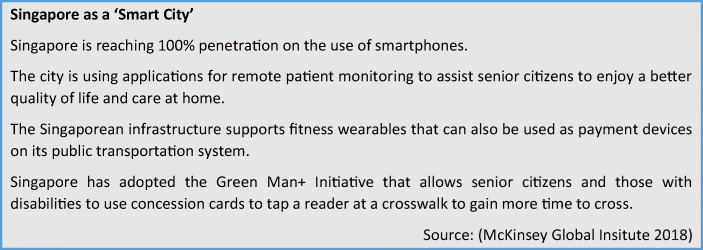

## Issues in Cross-Sectoral Data Sharing and Use

In this domain, challenges and issues arise because several key complex factors come together when these kinds of cross-sectoral data exchanges occur. These challenges include the following:

1. The fluid and apparently ever-expanding nature of what we mean by ‘health’, to include health-related matters, as well as well-being, welfare, the role of socio-economic and environmental influences and—potentially—moral agency itself, e.g. the importance of supporting and facilitating citizens’ and consumers’ choices.^2^ Thus, it becomes easier to make the case for seeking access to data across diverse sectors under a broad umbrella of health or health-related concerns. But this raises questions about the legitimacy of such requests and how these are to be assessed. For example, some requests for data may masquerade as health-related concerns while actually masking other motives, such as financial gain.
2. The considerable diversity of standards, regulations and codes of conduct (or lack thereof) across multiple sectors. Moreover, there are potentially divergent and/or conflicting drivers of action between any two sectors when there is a proposal to share or transfer data between sectors. This can have implications both for the adequate protection of citizens’ rights and interests, as well as for the respective stakeholders within sectors. For example, data controllers might have little reason or motivation to share data, and researchers might face multiple and repeated barriers to effective data sharing.
3. The difficulty (if not impossibility) from the citizen’s perspective of tracing what happens to their data and to know who has access and for which purposes. Eventual uses and purposes might be very different to those for which data were originally provided. Raw ‘data’ generated in one context might be meaningless on its own, but when transferred to another environment and linked to other data, it might become meaningful *information*, the distinction here being that ‘information is data that are endowed with meaning and purpose’ (Drucker 2006). This information can then be used to take decisions that affect citizens in diverse ways. This has multiple implications. Some of the most concerning include whether citizens can give adequately informed consent to cross-sectoral data sharing, whether adequate privacy protection can be assured across diverse and multiple sectors, whether and how data users can be confident that there is social licence for downstream uses that citizens might never foresee or anticipate, and whom citizens should trust in such complex and overlapping environments. The challenges of seeking to secure social licence are examined, for instance, in Ballantyne and Stewart (2019) in this issue of ABR and in Lawler et al. (2018).
4. The trust issues that data controllers themselves can experience when faced with access requests to datasets for which they are responsible. Data controllers might have legitimate concerns about the quality of their own data when releasing them for uses beyond their core purposes. Issues around quality are addressed in Lipworth (2019) in this issue of ABR. However, there might also be reputational concerns about creating new associations with parties in other sectors where security and governance mechanisms are unfamiliar or unclear. There will also always be potential liability concerns for data controllers as those with the ultimate legal responsibility for data and how it is processed. Matters are complicated further when multiple data controllers have to interact, especially across different legal systems, and also when new data controllers are created with the generation of novel datasets that contain (potentially) identifiable personal data.
5. From the perspective of researchers, the issue about the quality of datasets held within diverse sectors is also crucial. This can be compounded by the absence of quality metadata, i.e. data about the data that are held. Without robust metadata to make sense of datasets and to allow interoperability between datasets, effective cross-sectoral data sharing cannot happen. There can also be a significant lack of confidence in the efficiency and proportionality with which cross-sectoral governance mechanisms are run. A lack of co-ordination between sectors can lead to a duplication of access procedures, significant delays and the real risk of lost research investment for little or no gain in socially valuable research.

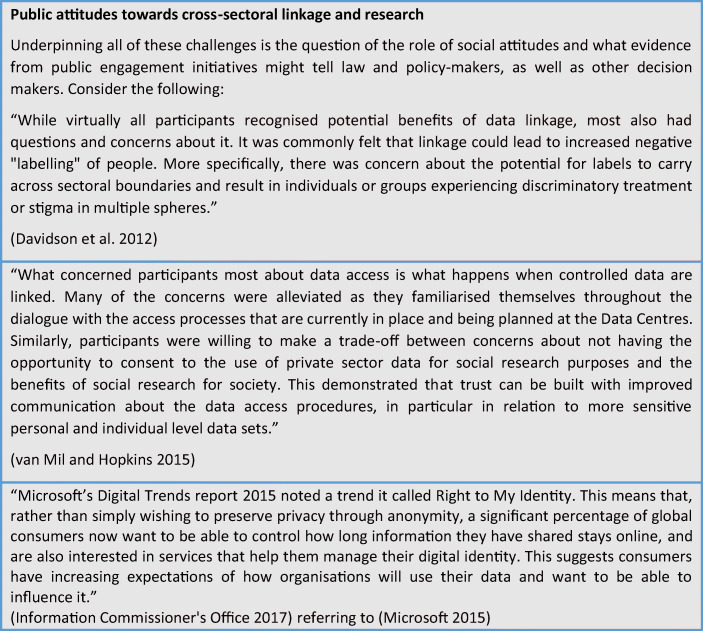

## Relevant Values

Most of the substantive and procedural values in this framework apply to the cross-sectoral context. *Privacy*, in particular, is a key concern as data flows from one sector to another, and the main ethical issues are addressed in the paper Openness in Big Data and Data Repositories (Xafis and Labude 2019) in this special issue. Similarly, as shown by some of the examples above, cross-sectoral big data initiatives raise serious questions about the viability of *consent* and *anonymization* as robust governance mechanisms to sufficiently protect citizens in this context. These limits are addressed in the section on *Inability to rely solely on data masking techniques and de-identification* in Xafis et al. (2019). Here, the discussion concentrates on the more distinctive issues and values engaged by cross-sectoral big data.

The substantive values of most relevance are as follows:

- *Privacy*: there are myriad threats to citizens’ privacy in the cross-sectoral context. Examples include the fact that different laws, regulations and standards of protection will apply to data within different ecosystems; moreover, with the intense accumulation of data across diverse aspects of people’s lives adverse decisions might be taken about them about which they know nothing and over which they have no control.
- The need to reflect upon and articulate the *public benefit* that might likely arise from cross-sectoral data sharing and use. While direct health benefits to citizens are often self-evident, the public benefits of objectives that are grounded in ‘well-being’ or ‘choice’ might require deeper reflection and robust justification. This is especially so when offset against other considerations, such as increased privacy risks from cross-sectoral data sharing and/or the limits or absence of consent as a basis for such sharing. Some organizations might be set-up entirely to process data on the basis of consent at the point of data collection; as such, cross-sectoral linkage might not be possible. However, *public interest* is often used as an alternative legitimate ethical and legal basis for data sharing, but its meaning is context-specific and requires clear articulation and justification.
- The complexities of cross-sectoral data use require consideration of the role and importance of *proportionality*. Whenever possible, governance and decision-making regimes should provide adequate protection of citizens’ claims and interests while also seeking to promote access and use that has demonstrable public benefit. As such, the balance to be struck is in ensuring that procedures for sharing and accessing data are proportionate to the risks involved and the benefits that are sought. Burden minimization and risk minimization are twin considerations.
- *Stewardship* is also concerned with the twin objectives of taking care of the object of attention—such as data—as well as seeking actively to promote its utility. The value of stewardship involves guiding others with prudence and care across one or more sectors with a view to collective betterment.

The most relevant procedural values are as follows:

- *Transparency*, which in this context suggests that both citizens and data controllers must be able to know what is done with their data and to the trace its uses, within practicable limits. The details of cross-sectoral initiatives should be open and accessible, including specifics about which datasets are being used (for data subjects) and which are available (for data users and researchers).
- *Reasonableness*, which is important because data uses might go well beyond the original purposes for which data were obtained or provided. In all cases, data subjects must be provided with good reasons to accept novel data sharing and use. Here good reasons must be those that are widely recognized as relevant and fair. This is so even when this might be in circumstances where they personally object. Any measures to opt out should be easily knowable, in line with the value of transparency.
- *Accountability*, which can be difficult to trace in the cross-sectoral context. For this reason, and once again reflecting the above values, it is all the more important that lines of responsibility for decision-making are clear, and means to hold decision-makers responsible are also part of the requirements of this value.
- *Engagement* of all stakeholders—from data subjects to researchers to the range of data controllers involved—which is robustly conducted will be a hallmark of an ethically robust ecosystem for cross-sectoral data sharing and use.
- *Trustworthiness*, which emphasizes both the need for individuals and institutions to demonstrate being worthy of trust, as well as relating to the nature of data available for sharing (quality), and of the access and oversight mechanisms (governance).

## Case Study: Deciding Whether and How To Share Data Across Data Ecosystems

The following hypothetical example illustrates how the values above might be used to help stakeholders involved in a prospective cross-sectoral data linking initiative to think through the issues and to decide and justify how this might be designed and delivered.

- *Scenario*: Caring well for an increasingly aged population is one of the most pressing challenges that governments face today. The Veteris Foundation is a non-profit charitable organization that has been working for a decade to address some of these challenges. It is keen to explore the opportunities provided by the IoMT. The Foundation has been in partnership with a wearable device manufacturer to develop app software and device sensors to the stage where they are confident that the data are accurately captured. Data are stored on the Veteris Foundation’s own server, to which the device manufacturer also has access. The data that are stored include heart rate, blood pressure, cholesterol, blood sugar levels, breathing rate, body temperature, food intake, weight, and personalized movement and exercise tracking. Much more data could be held, and many more parties, such as other apps or devices with a focus on health and fitness, could have access. App ‘families’ can interact and share data, and Bluetooth can be used to stream data continuously, e.g. heart rate or blood pressure. In turn, this kind of data could be used to raise alerts in emergency situations for wearers.

The Veteris Foundation only permits third parties, such as device developers, to access data via their server. They use a public application programming interface to facilitate this. As a result, the third parties receive all data generated by their respective devices. This enables data mining at an aggregate analysis of data (that is, at population level) and further downstream uses of data. Wearable device users do not know what is done with these data.

The Veteris Foundation sees tremendous potential to address the social challenge of an ageing population through its app and associated wearable device. It develops a five-level strategy:

- To explore linkage of the app and device to electronic health records (EHRs)
- To make the new dataset openly available for further research into ageing
- To offer the device free to groups aged 65+ that will support better care at home
- To make the health and mobility data available to social services to improve efficiency in community care, such as concentrating on day care centres for the elderly that can provide some respite cover for carers. In turn, Veteris will seek access to social care records in order to improve its own algorithms
- To seek new linkages with other datasets beyond those that it holds in order to further enrich its data resources and their potential

### Who Are the Stakeholders?

The first challenge that the Veteris Foundation will face is to establish which individuals and institutions are implicated in this kind of cross-sectoral data sharing initiative. EHRs will clearly be held within the healthcare system, possibly with ultimate authority sitting with a government body or agency, such as a Ministry of Health (or equivalent). Social care and community care initiatives will be the responsibility of different public authorities, and data controllers will exist in each sector with ultimate responsibility over data protection. However, institutions and organizations can only speak to their own data; they cannot anticipate in advance what might be considered ‘health-related’ by a third party, nor on what basis access and linkage might be sought. This poses major challenges for preparedness of datasets and organizations in making themselves safely and ethically open for cross-sectoral data sharing and linkage.

The values of *transparency*, *accountability* and *engagement* suggest that each of the relevant parties be involved in planning at the earliest possible stage—ideally collaboratively—to co-produce plans on how any cross-sectoral linkage might work and also to reveal the full range of ethical issues. All final plans should be publicly available, ideally for consultation.

### Community Involvement

Given the focus on the elderly community, the value of *engagement* also suggests that its members, as well as their carers and wider support groups, should have the opportunity to be involved in the proposal. This is particularly important because of the large-scale nature of what is envisioned. For example, how will the value of *privacy* be adequately protected when data are shared between different sectors? How far will the initiative respect citizens’ *autonomy* if data collected for one purpose might be used for a different purpose? Who might have access to data and for which purposes? What assurances can be given that could address any concerns that arise out of a public *engagement* exercise?

### Applying the Deliberative Balancing Approach—Step 1: What Exactly Is at Stake?

It will be crucial for the Veteris Foundation to be able to explain the technology behind the proposal in understandable terms, as well as their motivations in pursuing these activities, and to demonstrate that the cross-sectoral project has a reasonable likelihood of *public benefit*. An important fundamental issue to address is what has been called ‘the nexus of ethics and methodology’ (Nicholls et al. 2016). Put simply: do these apps and devices actually produce data of sufficient quality and are the linkage methods and protocols fit-for-purpose to deliver personal and public benefit to citizens? Does the necessary metadata exist to support linkage, and if not, how is this to be produced before plans impact on citizens?

If citizens are to be offered devices for free, are their choices to accept sufficiently informed and free from coercion? The value of *liberty* is important here.

If data are to be linked and shared, will this only be aggregate data (population level) or are there increased risks of individuals or groups being identifiable, both at the moment of initial linkage and over time as new linkages are made? How are *privacy* risks set-off against likely *public benefit*, especially when this benefit might be less directly about citizens’ health? Are improvements in efficiency for the delivery of social care good enough reasons?

How can stakeholder institutions that enter into a cross-sectoral data sharing initiative continue to demonstrate their *trustworthiness*?

### Step 2: How Do the Framework Substantive Values Help the Project?

The importance of demonstrating a reasonable likelihood of *public benefit*, justifiably offset with risks to *privacy*, *autonomy* and other individual interests, has been stressed above. Reliance on consent in this context might be challenging for numerous reasons, including the difficulty of explaining what might be done with data and by whom; potential issues of effective communication with an elderly group, and the difficulty for both citizens and consent takers of knowing which family of apps might be linked and using data.

Given the multiplicity of actors and data controllers across diverse sectors, the importance of the values of *transparency* and demonstrating *trustworthiness* becomes particularly acute.

The value of *proportionality* in this context is also crucial. It is likely, for example, that each sector will have its own system of information governance, and each might include separate processes for granting access to datasets and allowing sharing and use. Different standards might be in operation, including security and technical standards and protocols to enable the exchange of data with a range of servers. The technical challenges are not insurmountable^3^ (however, the ethics and governance hurdles also need to be considered for their proportionality. For example, are there ways to agree among gatekeepers, such as access committees, that only one (or a few) approvals are sufficient across all sectors? Is it possible to agree a common set of values to be taken into consideration when assessing applications for data linkage or use?

*Stewardship* also has a central role to play in this kind of scenario. Do sector partners already have experience of data linkage and sharing, and if so what lessons might be learned? Are there individuals within organizations who can take on a stewardship role to help broker acceptable arrangements for data access and use? Might organizations agree among themselves that a particular institutional body, such as a data access committee, can take on a meta-role for the cross-sectoral initiative? More work is required on the ethics of being a data steward, as opposed to a data controller (and the two are not mutually exclusive). While the role of the latter is enshrined in many legal systems as a legal responsibility to ensure compliance with data protection and other laws, a data steward has both a protecting and a promotion role with respect to the data for which they are responsible. Here ‘promotion’ refers to responsible further use of data towards justifiable ends; in contrast, a culture of caution within organizations or sectors can hinder full realization of this stewardship role.

### Step 3: What Are Likely Actions and How Might the Project Engage Dissenting or Sceptical Voices?

Doing nothing is always an option, but it is not a value-free decision. In exploring what could be done, engagement with all stakeholders will be crucial, and it is highly likely that any *engagement* exercise will produce some dissenting voices, both from citizens and data controllers. Some citizens might disapprove of this kind of initiative because it generates a sense of losing control over their data and their identity. Here, the value of *reasonableness* might help to explore the concerns and to identify the range of good reasons why the project might nonetheless proceed, with appropriate reassurances and safeguards.

Data controllers might legitimately be concerned to become involved because of worries about data quality and/or reputation risks and/or potential liability. Here, the value of *public benefit* can help such institutions organize for preparedness for sharing. In such a complex set of overlapping environments, it is inevitable that different sectors will be at different stages of preparedness. It is also important that any governance mechanisms are kept under regular review—perhaps subject to independent oversight—and to ensure that a range of perspectives continue to be taken into account.

### Steps 4 and 5: Giving Effect to the Deliberative Balancing Approach of This Framework

In giving effect to the balancing approach advocated in this framework, ethical *reflexivity* can assist greatly to identify the *kinds* of challenges being faced, and also in assessing whether a particular organization is ready to engage in cross-sectoral data sharing. This is likely to be one of the major hurdles to this activity. Reflexivity involves processes of self-reference of examination and action, requiring institutions and actors to look back at their own practices, successes and failures, which in this context will involve examining its ethical positions regarding data sharing and whether these need reconsideration.

Thus, for the cross-sectoral context, it is vital to distinguish between real and perceived challenges and to assess the state of preparedness of an institution to engage in robust data re-use. Reflexive thinking can:

(i) Clarify which considerations are purely legal, a mixture of legal and ethical and/or related to organizational culture
(ii) Expose those concerns which are perpetuated by legal myths or perceived controversy as opposed to those that can be adequately addressed within a proportionate governance framework for data sharing and use
(iii) Acknowledge an often-neglected element, namely, the problematic organizational and behavioural dynamic around data^4^

**Diagram 1:**
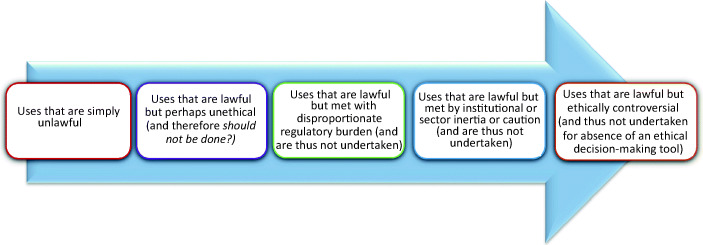
Assessment of real and perceived challenges in data re-use

Diagram 1 helps decision-makers to diagnose what might be at stake

Accordingly, while a particular organization or institution might not—yet—be ready to share or use data in a cross-sectoral environment, these ethical processes, based on the substantive and procedural values of this framework, can assist for the future. Assuming sufficient preparedness to engage in cross-sectoral sharing, then the five steps of the balancing approach can be used to weigh up the relative ethical merit of the different options being proposed.

In the above example of the Veteris Foundation, each element of its strategy should be subjected to this process in light of as much evidence is available about the likelihood of benefits, risks and harms, as well as an identification and assessment of the robustness of governance arrangements that would be in place to oversee the cross-sectoral activities.

### Step 6: Communication of the Outcome

Whatever the outcome of these deliberations, step 6 of the framework requires that the eventual decision be communicated transparently to all stakeholders. Self-evidently, if a decision is taken to proceed with cross-sectoral data sharing, this will be more challenging with regard to informing publics across sectors. While there is no ethical or legal requirement necessarily to attempt to inform individuals directly, there should be as a minimum privacy notices made available on websites and in locales where citizens would expect their data to be collected and used informing them of wider usage.

## Conclusion

The McKinsey *Report on Smart Cities* (McKinsey Global Institute 2018), referred to above, suggested that Singapore might be on the path to a ‘virtuous cycle of benefits’ from becoming a smart city. The values and approach of this ethical framework demonstrate that robust ethical reflection and action are central to the success of any such initiative.

## Footnotes

1 A review of literature on the ethical issues can be found in Mittelstadt (2017).

2 Cf. World Health Organization definition of health: ‘Health is a state of complete physical, mental and social well-being and not merely the absence of disease or infirmity’ (International Health Conference 2002).

3 As an illustration, see (Gay and Leijdekkers 2015).

4 Adapted from (Laurie and Stevens 2016).
